# Neutralizing Activity and SARS-CoV-2 Vaccine mRNA Persistence in Serum and Breastmilk After BNT162b2 Vaccination in Lactating Women

**DOI:** 10.3389/fimmu.2021.783975

**Published:** 2022-01-11

**Authors:** Kee Thai Yeo, Wan Ni Chia, Chee Wah Tan, Chengsi Ong, Joo Guan Yeo, Jinyan Zhang, Su Li Poh, Amanda Jin Mei Lim, Kirsten Hui Zhi Sim, Nursyuhadah Sutamam, Camillus Jian Hui Chua, Salvatore Albani, Lin-Fa Wang, Mei Chien Chua

**Affiliations:** ^1^ Department of Neonatology, KK Women’s & Children’s Hospital, Singapore, Singapore; ^2^ Translational Immunology Institute, Singhealth Duke-NUS Academic Medical Centre, Singapore, Singapore; ^3^ Paediatrics Academic Clinical Programme, Duke-NUS Medical School, Singapore, Singapore; ^4^ Programme in Emerging Infectious Diseases, Duke-NUS Medical School, Singapore, Singapore; ^5^ KK Human Milk Bank, KK Women’s & Children’s Hospital, Singapore, Singapore; ^6^ Department of Paediatrics, KK Women’s & Children’s Hospital, Singapore, Singapore; ^7^ Singhealth Duke-NUS Global Health Institute, Singhealth Duke-NUS Academic Medical Centre, Singapore, Singapore; ^8^ Lee Kong Chian School of Medicine, Singapore, Singapore

**Keywords:** SARS-CoV-2 vaccine, mRNA vaccine, BNT162 vaccine, neutralizing antibodies, COVID-19, COVID-19 serological testing, breast feeding, breast milk expression

## Abstract

**Background:**

There is limited information on the functional neutralizing capabilities of breastmilk SARS-CoV-2-specific antibodies and the potential adulteration of breastmilk with vaccine mRNA after SARS-CoV-2 mRNA vaccination.

**Methods:**

We conducted a prospective cohort study of lactating healthcare workers who received the BNT162b2 vaccine and their infants. The presence of SARS-CoV-2 neutralizing antibodies, antibody isotypes (IgG, IgA, IgM) and intact mRNA in serum and breastmilk was evaluated at multiple time points using a surrogate neutralizing assay, ELISA, and PCR, over a 6 week period of the two-dose vaccination given 21 days apart.

**Results:**

Thirty-five lactating mothers, median age 34 years (IQR 32-36), were included. All had detectable neutralizing antibodies in the serum immediately before dose 2, with significant increase in neutralizing antibody levels 7 days after this dose [median 168.4 IU/ml (IQR 100.7-288.5) compared to 2753.0 IU/ml (IQR 1627.0-4712.0), p <0.001]. Through the two vaccine doses, all mothers had detectable IgG1, IgA and IgM isotypes in their serum, with a notable increase in all three antibody isotypes after dose 2, especially IgG1 levels. Neutralizing antibodies were detected in majority of breastmilk samples a week after dose 2 [median 13.4 IU/ml (IQR 7.0-28.7)], with persistence of these antibodies up to 3 weeks after. Post the second vaccine dose, all (35/35, 100%) mothers had detectable breastmilk SARS-CoV-2 spike RBD-specific IgG1 and IgA antibody and 32/35 (88.6%) mothers with IgM. Transient, low intact vaccine mRNA levels was detected in 20/74 (27%) serum samples from 21 mothers, and 5/309 (2%) breastmilk samples from 4 mothers within 1 weeks of vaccine dose. Five infants, median age 8 months (IQR 7-16), were also recruited - none had detectable neutralizing antibodies or vaccine mRNA in their serum.

**Conclusion:**

Majority of lactating mothers had detectable SARS-CoV-2 antibody isotypes and neutralizing antibodies in serum and breastmilk, especially after dose 2 of BNT162b2 vaccination. Transient, low levels of vaccine mRNA were detected in the serum of vaccinated mothers with occasional transfer to their breastmilk, but we did not detect evidence of infant sensitization. Importantly, the presence of breastmilk neutralising antibodies likely provides a foundation for passive immunisation of the breastmilk-fed infant.

## Introduction

Coronavirus disease 2019 (COVID-19) messenger RNA (mRNA) vaccines have been increasingly deployed in many countries as a means of controlling infectious spread and severity of the disease (1–4). Even so, the initial clinical trials evaluating these novel mRNA vaccines, encoding the spike protein of the severe acute respiratory syndrome coronavirus 2 (SARS-CoV-2), excluded breastfeeding and lactating women (4, 5). There is limited current data on the efficacy and safety of these SARS-CoV-2 vaccines in this group of mothers and their breastmilk-fed infants (6–8).

Emerging evidence from several cohort studies on lactating women have demonstrated the immunogenicity of the currently available mRNA vaccines (BNT162b2, mRNA-1273) among lactating women, with the induction of SARS-CoV-2-specific antibodies in the breastmilk post-vaccination (9–14). However, there is paucity of information on the functional neutralizing capabilities of SARS-CoV-2-specific antibodies in breastmilk and their dynamic and temporal relationship to serum levels after mRNA vaccination. Additionally, the potential adulteration of breastmilk with vaccine mRNA is currently unknown and raises safety concerns relating to the potential exposure of breastmilk-fed infant to the mRNA. Several international organizations including the World Health Organization (15) have recommended the continuation of breastmilk feeding following vaccination, while acknowledging the lack of safety data for mother and child.

To address these issues, we investigated the dynamics of SARS-CoV-2-specific immunoglobulin subtypes and their temporal relationship with SARS-CoV-2 neutralizing activity in the serum and breastmilk of lactating mothers through the 2-dose BNT162b2 mRNA vaccine, and the post-vaccination persistence of vaccine mRNA in the serum and breastmilk of these vaccinated mothers. We also examined serum of breastmilk-fed infants from vaccinated mothers to determine the presence of SARS-CoV-2 neutralizing antibodies and vaccine mRNA.

## Materials and Methods

### Study Population

We evaluated the humoral responses of a cohort of healthcare workers who were lactating mothers working at a tertiary level women’s and children’s hospital in Singapore and had received the BNT162b2 COVID-19 vaccine (Pfizer/BioNTech) between 15 January and 31 May 2021. These front-line healthcare workers, were eligible if they consented to blood and breastmilk collection at specific timepoints after vaccination. All participants received both vaccine doses (30 μg/0.3 ml) 21 days apart. Breastmilk-fed infants from these lactating mothers were also recruited for the collection of a single serum sample with informed consent. At enrolment, maternal and infant demographic and clinical information were collected, including any significant symptoms after any of the two vaccine doses. The study was approved by the Singhealth Institutional Review Board and all participants provided written informed consent (CIRB Ref. No 2019/2906 & CIRB Ref. No. 2016/2791).

### Biological Samples

Breastmilk samples (10mls each) were collected on day of vaccination (day 0) followed by days 1, 3, 7, 14, and 21 post-vaccination for both doses. Breastmilk sample on day 21 after dose 1 was collected before receipt of dose 2. All mothers were advised to express breastmilk into sterile containers and to immediately store them in their own freezers before transportation to the laboratory in coolers containing frozen cold packs. For the initial processing of these breastmilk samples, they were thawed and centrifuged twice at 2383g for 15minutes at 4°C. The fat layer was removed after each spin cycle and the resultant skim milk was transferred to a cryovial and frozen at -80°C until analysis.

Maternal serum samples were obtained at days 0 and 3 for dose 1 and days 0 and 7 for dose 2. Samples on day 0 of dose 2 was obtained before vaccine was administered. Infant serum samples were collected >3 weeks post maternal second dose. All serum samples (0.5ml to 1ml) were collected in serum separation blood collection tubes (Sarstedt AG & Co, Germany) and transported to the laboratory on the same day. These samples were centrifuged at 1300g for 10 minutes before serum was aliquoted into cryovials and stored at -80°C until analysis.

### SARS-CoV-2 Surrogate Viral Neutralization Assay

We utilized a SARS-CoV-2 surrogate virus neutralization assay (cPass™ SARS-CoV-2 Neutralization Antibody Detection Kit, GenScript Inc., USA) that detects the total immunodominant neutralizing antibodies targeting the viral spike protein receptor binding domain (RBD) in an isotype and species independent manner (16). This validated and commercialized test developed with the D614 SARS-CoV-2 strain, measures the magnitude of antibody-mediated blockage of the interaction between angiotensin-converting enzyme 2 (ACE2) receptor protein and the RBD of SARS-CoV-2 which is required for viral entry into susceptible cells (17). Prior studies have documented that RBD-targeting neutralizing antibodies are immunodominant with SARS-CoV-2 infections (18, 19). The cPass kit results have shown 95.7% positive predictive agreement (95%CI 85.8-98.8%) and 97.8% negative predictive agreement (95%CI 92.5 – 99.4%) with 50% viral neutralization by plaque reduction neutralization tests (PRNT) in clinical studies (16, 20).

For serum samples, a final dilution of 1:20 was used according to manufacturer’s recommendations. As the test was only validated on serum/plasma by the manufacturer, an optimization was performed and adapted for breastmilk samples. A final dilution of 1:5 was used as it gave no false positive background on pre-vaccinated breastmilk samples and allowed maximum volume of breastmilk to be tested. Apart from the sample amount used for testing, the rest of the assay was performed per manufacturer’s instructions. The percent signal inhibition was calculated = [1-(Optical Density (OD) of sample/OD of negative control)]x100%. An inhibition signal of ≥30% was used as the cutoff for positive detection of SARS-CoV-2 neutralizing antibodies in all sample types (20).

Inhibition signal from individual samples from the cPass assay was converted to the World Health Organization (WHO) International Units (IU) based on previous calibration of this neutralization assay against the WHO International Standard (IS) for SARS-CoV-2 neutralization assays (21, 22), using a Excel-based conversion tool available online (https://github.com/Lelouchzhu/cPass-to-IU_Conversion). Based on recent studies using biological replicates from different international groups, cPass readings (% inhibition) were shown to be highly reproducible to International Units (IU)/ml of the WHO International Standard, with a pseudo R^2^ at 0.978. cPass inhibition signal of 30% corresponds to a cut-off of 28 IU/ml for serum samples and 7 IU/ml for breastmilk samples based on the conversion to WHO International Standard and dilution factor for sample type.

### Enzyme-Linked Immunosorbent Assay for Detection of SARS-CoV-2 Antibody Isotype

To determine the relative abundance of SARS-CoV-2 antibody isotypes and compare their temporal dynamics with neutralization activity, we performed semi-quantitative evaluations of the SARS-CoV-2 spike protein receptor binding domain (RBD)-specific antibody isotypes in serum and milk by enzyme-linked immunosorbent assay (ELISA). The 96-well plates were coated with 2μg/mL of SARS-CoV-2 RBD protein (RBD-His tag, expressed in HD293F cells from Genscript, USA) (100ng protein/well) diluted in bicarbonate buffer at 50μl/well in 4°C overnight. Plates were washed with wash buffer (0.05% Tween 20 in 1×DPBS) and blocked with 150μL of blocking buffer (BD OptEIA Assay Diluent, BD Pharmingen, USA). Block solution was discarded and plates were blotted dry. Serum diluted 1:100 in blocking buffer or neat breastmilk were added (50μl per well) and incubated for 2 hours at room temperature. Naïve human serum samples were added to each plate as negative controls.

This was followed by 5 more washes with wash buffer and incubated for 1-hour at room temperature with 50μl per well of 1μg/ml mouse anti-human IgG1 to IgG4 (Southern Biotech, USA), mouse anti-human IgM (GenScript Inc., USA) and goat anti-human IgA (Southern Biotech, USA). Plates were washed 5 times with wash buffer and underwent 1-hour incubation at room temperature with 50μl per well of 1:10000 anti-mouse IgG horseradish peroxidase (HRP) (Biolegend, USA) for detection of IgG1 to IgG4 and IgM isotypes, and 1:10000 anti-goat IgG-HRP (Biolegend, USA) for detection of IgA. Plates were washed 5 times with wash buffer and 50μl of TMB ELISA substrate (Life Technologies, USA) were added per well. Plate development was stopped by addition of 50μl KPL TMB Stop Solution (Sera Care, USA). Absorbance on the BioTek Cytation5 plate reader (Fisher Healthcare, USA) at 450nm and a reference background of 570nm was recorded. Corrected absorbance value was calculated (450nm-570nm). Corrected Optical Density at 450nm (OD_450_) values for individual sample and isotypes was obtained by subtracting value of negative control of each isotype from each individual plate.

### BNT162b2 mRNA Detection

RNA from breastmilk and serum samples was extracted using the E.Z.N.A Total RNA extraction kit (Omega Bio-tek Inc., USA) according to manufacturer’s instructions. Briefly, samples were treated with lysis buffer and ethanol before binding of RNA to the HiBind RNA Mini Column. After several washes with wash buffer, RNA was eluted into nuclease-free water and immediately stored at -80°C before analysis. Synthesis of complementary DNA was performed using QuantiTect Reverse Transcription kit (Qiagen NV, Germany) and Real-time PCR was performed using SensiFAST SYBR No-ROX kit (Meridian Bioscience, USA). Primers were designed using the published BNT162b2 mRNA sequence (23). The following forward primer 5’ TTCGCCCAAGTGAAGCAGAT 3’ and reverse primer 5’ CGGCCAGTGTCACTTTGTTG 3’ with annealing temperature of 60°C was used. All samples were run in triplicates and repeated for result confirmation. Purified BNT162b2 mRNA was used as standard for quantification. Standard curves were generated for individual runs using spiked vaccine mRNA into non-vaccinated samples (see **Supplemental Figure 1**).

### Statistical Analysis

Data generated are presented as median with interquartile range (IQR). Comparisons of median values of samples were performed using Mann Whitney U test. Correlation analysis was performed with Pearson correlation coefficients. Comparison of antibody isotype levels from pre-vaccination to the multiple post-vaccination timepoints were assessed using repeated measures mixed-effects model followed by *post hoc* Tukey’s multiple comparisons test. Statistical significance was defined as p<0.05 and was two tailed. All statistical analysis was performed using GraphPad Prism 9 (GraphPad Software, USA).

## Results

### Study Population

We enrolled 35 lactating mothers who were frontline healthcare workers and received the two-dose BNT162b2 vaccine. Thirty-one women were recruited before their first dose and 4 were included just before their second dose. All participants completed the 2-dose course within 21 days. These mothers had a median age of 34 years (IQR 32-36), were predominantly of Chinese ethnicity (74%) and all had full-term deliveries (**Table 1**). The median age of their child and the length of lactation at the first vaccine dose was 7 months (IQR 5-14). All mothers were breastfeeding and/or feeding expressed breastmilk to their child. Five infants, with median age 8 months (IQR 7-16), were recruited into this study and provided serum samples. No participants were diagnosed with COVID-19 before or during the study period and none reported significant allergic symptoms with the vaccination.

**Table 1:** Clinical characteristics of the lactating mothers.

Characteristics	Total (n=35)
Median maternal age at first dose, years (IQR)	34 (32 – 36)
Maternal ethnicity, n (%)	
Chinese	26 (74)
Malay	5 (14)
Indian	1 (3)
Other	3 (9)
Median child gestation at birth, weeks (IQR)	39 (38 – 39)
Female child, (%)	22 (63)
Median age of child at maternal first dose, months (IQR)	7 (5 – 14)
Predominant mode of feeding, n (%)	
Breastfeeding	12 (34)
Expressed breast milk	17 (49)
Both breastfeeding + expressed breast milk	6 (17)
Estimated average volume of breastmilk per day, ml (range)	550 (190 – 1000)
Reported side effects after vaccine doses, n (%):	
Nil side effects	10 (29)
Myalgia	15 (43)
Fever	4 (11)
Rhinorrhea/Cough	3 (9)
Mastitis	1 (3)
Headache	1 (3)
Joint pain	1(3)

### Neutralizing Antibody Level

#### Serum of Vaccinated Mothers

**Figure 1:**
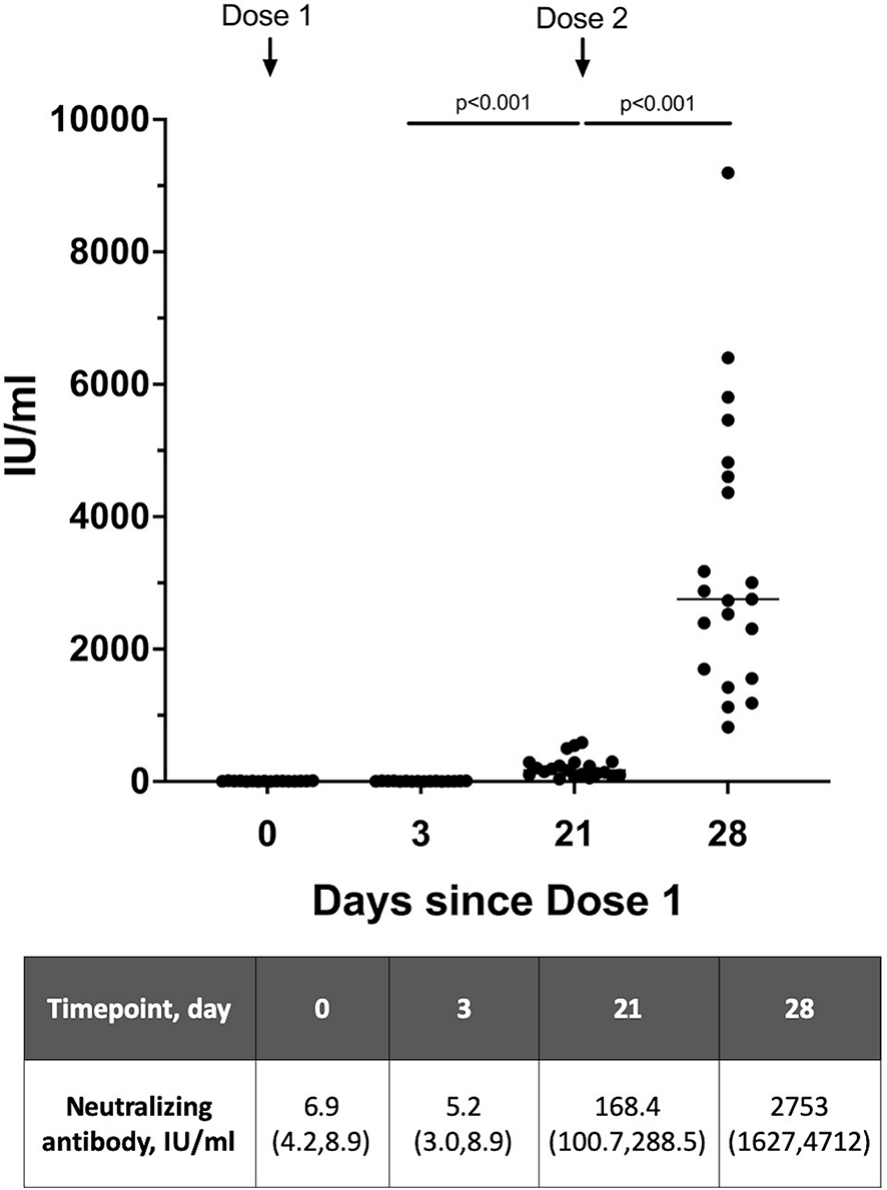
Neutralizing antibody levels detected in serum of lactating women over 2 vaccine doses expressed as WHO SARS-CoV-2 International Standard (n=21). Serum samples on day 21 is taken prior to receipt of Dose 2. A level >28 IU/ml was used as the cutoff for positive detection of neutralizing antibodies. Neutralizing antibody levels are presented as median (interquartile range), IU/ml.

To evaluate the neutralizing antibody responses from the 2-dose vaccination schedule in lactating women, a total of 21 mothers provided serum samples – 16 women provided 4 serum samples over 2 doses and 5 women provided 2 serum samples with the second dose. All women tested had detectable neutralizing antibody present in the serum just prior to the second dose (day 21) based on the 28 IU/ml cut-off (**Figure 1**). Median neutralizing antibody level at day 0-10 after the second dose (704.6 IU/ml, IQR 163.1-2784.0) was significantly higher than at day 0–3 after the first dose (6.4 IU/ml, IQR 3.4-8.9) (p<0.001). The median neutralizing antibody increased from dose 1 to dose 2 – with a rapid and significant increase in levels from day 0 to day 7-10 after vaccine dose 2 [median 168.4 IU/ml (IQR 100.7-288.5) vs 2753.0 IU/ml (IQR 1627.0-4712.0), p <0.001].

#### Breastmilk of Vaccinated Mothers

**Figure 2:**
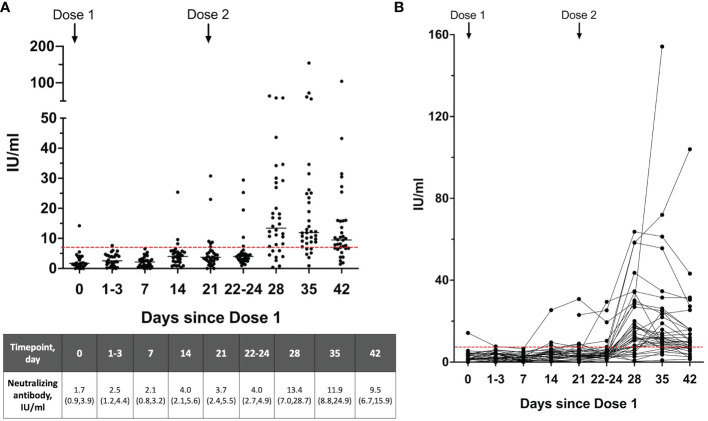
**(A)** Neutralizing antibody levels and the **(B)** dynamics of neutralizing antibodies detected in the breastmilk over the 2-dose BNT162b2 mRNA vaccination expressed as WHO SARS-CoV-2 International Standard (n=35). Breastmilk samples on day 21 is taken prior to receipt of Dose 2. A level >7 IU/ml (dotted red line) was used as the cutoff for positive detection of neutralizing antibodies. Neutralizing antibody levels are shown as median (interquartile range), IU/ml.

To determine the presence of neutralizing antibodies in breastmilk, 11 breastmilk samples per participant were collected from 31 women over the 2-dose vaccination and 6 samples per participant from 4 women with dose 2 of the vaccination schedule. There were minimal SARS-CoV-2 neutralizing antibodies present in the breastmilk (based on the 7 IU/ml cut-off) from day 0 of dose 1 up to day 3 post-dose 2 (**Figures 2A, B**). The neutralizing antibody levels increased significantly to a median of 13.4 (IQR 7.0-28.7) at 28 days (day 7 dose 2) from a median of 4.0 (IQR 2.7-4.9) at day 22-24 (p<0.001) (**Figure 2A**). Of those who provided breastmilk samples over 2 doses, only samples from 3 mothers did not have detectable neutralizing antibodies at any of the sampling timepoints up to 42 days [median 2.6 IU/ml (IQR 1.9-3.4)], in spite of these 3 mothers having detectable serum neutralizing antibodies.

**Figure 3:**
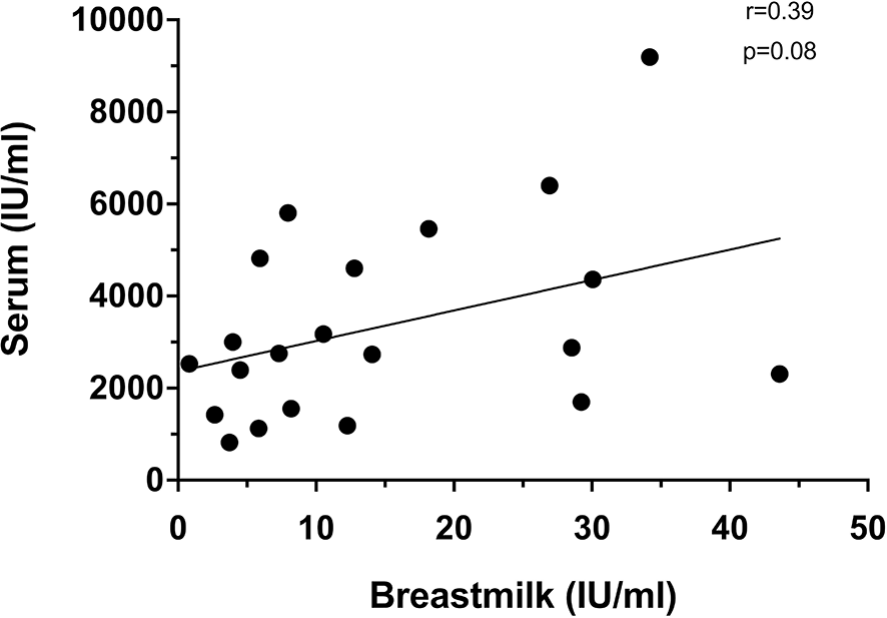
Correlation between serum and breastmilk neutralizing at day 7 after dose 2 (n=21).

Just prior to vaccine dose 2 (day 21), only 6/35(17.1%) mothers had detectable breastmilk neutralizing antibodies in spite of all mothers having detectable levels in their serum. After the second dose, there was moderate correlation between serum and breastmilk neutralizing antibody levels at day 7 dose 2 (r=0.39, p=0.08) (**Figure 3**). This was the timepoint with peak levels of breastmilk neutralizing antibodies detected for majority of mothers in this study.

#### Infant Serum

Five infants from the cohort of vaccinated mothers were recruited into the study and a single serum sample was collected from them. These samples were collected at a median of 48 days (IQR 44-57) after the second maternal vaccine dose. The age of these infants at the point of maternal vaccination ranged from 3 to 20 months. None had detectable neutralizing antibodies in their serum.

### SARS-CoV-2 Spike RBD-Specific Antibody Isotypes

#### Serum of Vaccinated Mothers

**Figure 4:**
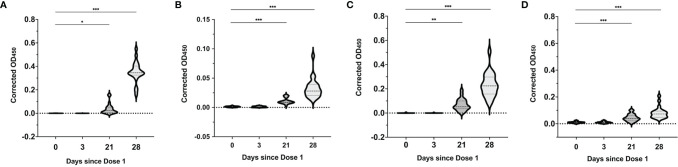
SARS-CoV-2 spike RBD-specific antibody responses in serum over the 2-dose BNT162b2 mRNA vaccination. Median serum corrected OD_450_ values over the different time points for **(A)** SARS-CoV-2 RBD-specific IgG1, **(B)** IgG3, **(C)** IgA and **(D)** IgM isotypes. Violin plots with included boxplots showing kernel probability density of corrected OD_450_ with the dashed and dotted black lines representing the median and 25th/75th quartiles respectively. Comparisons of differences between time points were assessed using repeated measures mixed-effects model followed by *post hoc* Tukey’s multiple comparisons test. The asterisk indicates P<0.05, double asterisk indicates P<0.001, the triple asterisk indicates P<0.0001.

Through the two vaccine doses, all mothers had detectable IgG, IgA and IgM isotypes in their serum with a notable progressive increase in all three antibody isotypes observed over the 4 study timepoints. There was significant boosting of the IgG1 levels 1 week after the second vaccine dose, with a 15-fold increase compared to day 21 after the first dose (median OD_450_ 0.02 (IQR 0.01-0.05) vs 0.35 (IQR0.33-0.40), P<0.0001) (**Figure 4A**). There was a lesser increment in the IgG3, IgA and IgM levels over the same timepoints (median OD_450_ 0.009 (IQR 0.008-0.014) vs 0.028 (IQR 0.02-0.04), p<0.0001), (median OD_450_ 0.05 (IQR 0.04-0.1) vs 0.22 (IQR 0.16-0.30), p<0.0001), and (median 0.04 (IQR 0.03-0.06) vs 0.07 (IQR 0.05-0.09), p<0.0001) (**Figures 4B**–**D**). IgG2 and IgG4 were not detected in the serum samples.

#### Breastmilk of Vaccinated Mothers

Up to day 21 of the first vaccine dose, SARS-CoV-2 spike RBD-specific IgG1 antibody were detected in the breastmilk of 23/31 (74.2%) mothers; IgA in 31/31 (100%) and IgM in 26/31 (83.9%) mothers. Post the second vaccine dose, all (35/35, 100%) mothers had detectable SARS-CoV-2 spike RBD-specific IgG1 and IgA antibody and 32/35 (88.6%) mothers with IgM.

**Figure 5:**
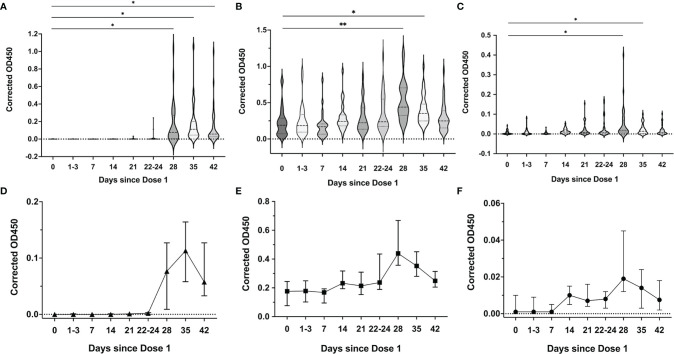
SARS-CoV-2 spike RBD-specific antibody responses in breastmilk over the 2-dose BNT162b2 mRNA vaccination. Median breastmilk corrected OD_450_ values over the different time points for **(A)** SARS-CoV-2 RBD-specific IgG1, **(B)** IgA and **(C)** IgM isotypes. Violin plots with included boxplots showing kernel probability density of corrected OD_450_ with the dashed and dotted black lines representing the median and 25th/75th quartiles respectively. Comparisons of differences between time points were assessed using repeated measures mixed-effects model followed by *post hoc* Tukey’s multiple comparisons test. Line graph depicting the dynamics of breastmilk (median with 95% CI as error bars) **(D)** IgG1, **(E)** IgA and **(F)** IgM isotypes over the time points. The asterisk indicates P<0.05, double asterisk indicates P<0.001.

Breastmilk IgG1 rose significantly 7 days after the second vaccine dose with continued persistence and elevated levels 21 days after (Day of dose 2: median OD_450_ 0.001 (IQR 0-0.002); 7 days after dose 2: 0.08 (IQR 0.004-0.3); day 14 after dose 2: 0.11 (IQR 0.05,0.2); day 21 after dose 2: 0.06 (IQR 0.03-0.2) (**Figures 5A, D**). IgA and IgM in breastmilk (**Figures 5B, C**) increased gradually to peak levels at day 7 post dose 2 (peak median IgA OD_450_ 0.4 (IQR 0.3-0.7), peak median IgM 0.02 (IQR 0.01-0.07). Levels of these IgA and IgM antibodies subsequently decrease to pre-dose 2 levels after 3 weeks (**Figures 5E, F**). IgG2, IgG3 and IgG4 subclasses were not detected in breastmilk samples.

#### Infant Serum

None of the five infant serum analysed had detectable SARS-CoV-2 spike RBD-specific IgG, IgM and IgA antibodies.

### BNT162b2 mRNA Detection in Serum and Breastmilk

**Figure 6:**
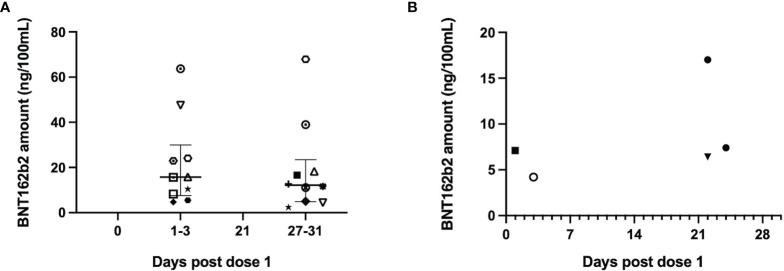
The BNT162b2 mRNA amount detected in maternal serum and breastmilk across the different timepoints. Amount of BNT162b2 mRNA (ng/100ml) in **(A)** serum and **(B)** breastmilk across the different sampling timepoints. Only positive samples are plotted and specific shapes in the plots denote samples from individual mothers across both sample types types (median and 25^th^/75^th^ quartiles plotted). Intact vaccine mRNA was detected in 20/74 serum samples tested from 21 mothers and 5/309 breastmilk samples tested from 31 mothers. Only 1 mother had detectable vaccine mRNA in serum and breastmilk samples (denoted by black filled square).

Vaccine mRNA was detected in 20 serum samples from 15 mothers, out of 74 samples from 21 mothers tested. A total of 10/16 (63%) and 10/25 (40%) mothers had detectable vaccine mRNA at day 1-3 of dose 1 and day 7-10 of dose 2 respectively (**Figure 6A**). Five mothers had positive serum samples at both time points. The median vaccine mRNA amount (ng/100ml) were not different between the two timepoints – 16 (IQR 9-24) compared to 12 (IQR 9-18) (p=0.6) (**Supplemental Table 1**). None of the samples on days 0 and 21 post-dose 1 had detectable vaccine mRNA.

Five breastmilk samples from 4 mothers had detectable vaccine mRNA, out of 309 samples from 31 mothers tested (**Supplemental Table 2**). All positive samples were collected within 3 days of the vaccine doses - two samples from days 1 and 3 of dose 1 (**Figure 6B**) and another three from days 1 and 3 post dose 2. One mother had detectable vaccine mRNA in both breastmilk and serum samples. The median vaccine mRNA amount in both sample types were comparable: 14ng/100ml (IQR 8-23) in serum compared to 7ng/100ml (IQR 6-7) in breastmilk (p=0.2).

None of the serum samples from the five infants tested had detectable vaccine mRNA. Of the five, one infant was from a mother with detectable vaccine mRNA in the both breastmilk and serum and another three were from mothers with vaccine mRNA in the serum.

## Discussion

In this study, we described the dynamics of SARS-CoV-2-specific antibody isotype production and the associated inhibitory activity in the serum and breastmilk of women vaccinated with the 2-dose BNT162b2 vaccine, over a 6-week period. As expected, there was detection of neutralizing antibodies and robust inhibitory responses in the serum just prior to and after the second vaccine dose, with all samples achieving significantly elevated neutralizing antibody levels by day 7 of this dose. The neutralization antibody levels and the associated SARS-CoV-2 RBD-specific antibody isotypes in the serum of these lactating women corresponds to previous reports of a rise in IgG, IgM and IgA specific to the spike and RBD segments of the SARS-CoV-2 virus after the first dose, and boosting of selected antibody isotypes by the second dose (10, 11). We similarly documented elevation in RBD-specific IgG1 and IgG3 levels in the serum of all mothers after the second vaccine dose. Recent studies have described the dominance of IgG1 and IgG3 response over IgG2 and IgG4 following SARS-CoV-2 mRNA vaccination and with natural infection (24–26). The lower IgM levels after the second vaccine dose may be related to enhanced class switching to IgG and IgA isotypes after this dose.

In the breastmilk, detection of neutralizing antibody and increase in inhibitory capability was notable 7 days after dose 2, corresponding with the peak levels of SARS-CoV-2 RBD-specific antibody isotypes, especially IgG1, and IgA levels at this point. These findings are concordant with previously reported increases in breastmilk SARS-CoV-2-specific IgA and IgG levels within a week after the second vaccine dose (10, 11, 27, 28). SARS-CoV-2 specific IgA in the breastmilk reportedly achieved maximal levels a week after the second dose, where SARS-CoV-2-specific IgG was only evident 1 week after the second dose (12, 14, 28–31). The persistence of neutralizing activity up to 3 weeks post dose 2 corresponds to the continued elevation in SARS-CoV-2 RBD-specific IgG1 but not IgA levels. In agreement with recent data, our study highlights the dominance of breastmilk SARS-CoV-2 IgG1 responses post-vaccination, as compared to higher IgA responses reported in natural infection (11, 30, 32, 33). The potential for therapeutic application of these antibodies for protection against COVID-19, especially neonatal infection, remains to be elucidated.

We noted one lactating mother who had detectable SARS-CoV-2-specific neutralizing antibodies in her breastmilk sample prior to and immediately after the first dose of BNT162b2 vaccination. There was no detectable neutralizing antibodies in her serum samples prior to and after the first vaccine dose (days 0 and 3), but with similar increases in the neutralizing antibodies after the second dose as the rest of the cohort. As this participant reported no significant COVID-19 exposure and the low community transmission of SARS-CoV-2 during the course of this study (34), this finding is likely due to cross-reactivity to antibodies from recent/past infection with non-SARS-CoV-2 coronavirus strains.

The neutralizing antibody levels in the serum and the breastmilk levels were poorly correlated prior to the second vaccine dose, with moderate positive correlation noted at a week after dose 2. This is likely a reflection of the boosting of antibody responses in the serum post the second dose and the enhanced excretion of antibodies into the breastmilk.

We also report the detection of low quantities of intact BNT162b2 mRNA in the serum and breastmilk samples – in 71% and 13% of the mothers investigated respectively. Several recent reports have inconsistently documented the presence of vaccine mRNA in the serum and breastmilk of women after BTN162b2 vaccination (13, 35, 36). The presence of intact vaccine mRNA in both sample types in our study highlights the stability and persistence of the vaccine mRNA nanoparticle within the bloodstream which may lead to infrequent transfer into breastmilk. The systemic spread of intramuscular delivery of lipid nanoparticle encapsulated-mRNA have been previously demonstrated in animal models (37). Importantly, our results were complemented by the lack of neutralizing antibodies and vaccine mRNA in the serum of breastmilk-fed infants from vaccinated mothers, suggesting the likely lack of significant exposure or sensitization of infant to the low levels of mRNA present in breastmilk. The concentration of mRNA detected in the serum and breastmilk are comparable, but the levels are still a small fraction of the vaccine dose given. The median concentration detected is 0.02% and 0.05% of the vaccine dose in 100ml of milk or serum respectively.

These results provide additional evidence on the immunogenicity of BNT162b2 mRNA vaccination among lactating women through the demonstration of robust SARS-CoV-2 neutralizing activity in the serum and breastmilk. Importantly, the presence of SARS-CoV-2-specific antibody isotypes and neutralizing antibodies in the breastmilk underscores the potential passive protection afforded to the breastmilk-fed infants. While we detected transient, low levels of intact vaccine mRNA in the serum and breastmilk, we did not detect any serological evidence of infant sensitization. These data provide additional support to the safety of current recommendations for continuing breastfeeding with maternal BTN162b2 mRNA vaccination.

The strengths of our study include the comprehensive, paired collection of serum and breastmilk samples at multiple collection timepoints over the 2-dose vaccine course which allowed us to investigate the presence and importantly, track the neutralizing ability of the antibodies present in both sample types. We utilized a rigorous detection method for the confirmation of the presence of low levels of intact vaccine mRNA in the serum and breastmilk. This study is limited by the convenience cohort of front-line healthcare workers and a small number of infants, which may limit generalizability to the general population. The positive detection of the vaccine mRNA in serum and breastmilk did not extend beyond 1 week after vaccination, although we were limited by the number of serum collection timepoints. Due to the self-collection and storage of breastmilk in the participants freezers prior to transport to the laboratory, this may have led to variation in sample quality which in turn could have led to degradation in the vaccine mRNA prior to detection (38).

Larger population-based study should be performed to confirm the persistence of intact vaccine mRNA in the serum and breastmilk of lactating women who received the SARS-CoV-2 vaccination and the possible sensitization of breastmilk-fed infants towards the intact mRNA. This will contribute additional evidence to support the safety of current and future recommendations for infant feeding with mRNA vaccination. Future studies should also further examine the SARS-CoV-2 inhibition activity of specific antibody isotypes present in breastmilk through mRNA vaccination, and the potential for infant protection against COVID-19 through the passive transfer of breastmilk-derived SARS-CoV-2-specific antibodies.

BNT162b2 mRNA vaccination was associated with the generation of robust SARS-CoV-2 neutralizing responses in the serum and breastmilk of lactating women. While we detected low levels of vaccine mRNA in the serum of vaccinated mothers and their breastmilk, we did not detect any serological evidence of sensitization of the infant towards the mRNA, providing additional support to the safety of current recommendations for continuing breastfeeding with maternal BTN162b2 mRNA vaccination.

## Data Availability Statement

The original contributions presented in the study are included in the article/**Supplementary Material**. Further inquiries can be directed to the corresponding author.

## Ethics Statement

The studies involving human participants were reviewed and approved by the Singhealth Institutional Review Board (CIRB ref. no 2019/2906 & CIRB ref. no. 2016/2791). Written informed consent to participate in this study was provided by the participant or by the participant’s parent/legal guardian for the infant.

## Author Contributions

Conceptualization: KY, WC, CT, JY, SA, L-FW, and MC. Recruitment of volunteers and laboratory samples collection: KY, CO, KS, and MC. Data acquisition, analysis and interpretation of data: KY, WC, CT, CO, JY, JZ, SP, AL, KS, NS, and CC. Drafting of the manuscript: KY, WC, CT, CO, and JY. Statistical analysis: KY and WC. Overall supervision: SA, L-FW, and MC. KY, WC, and CT had full access to all data in the study and take responsibility for the integrity and accuracy of the data presented. All authors critically revised the manuscript for intellectual content, approved the version of manuscript submitted and agree to be accountable for all aspects of the work.

## Funding

This study was conducted with the provision of grant funding from the SingHealth Duke-NUS Academic Medicine COVID-19 Rapid Response Research AM/COV004/2020 (SA), National Medical Research Council (NMRC) Centre Grant Programme (NMRC/CG/M003/2017) (SA), Singapore National Research Foundation (NRF2016NRF-NSFC002-013)(L-FW) and National Medical Research Council (STPRG-FY19-001, COVID19RF-001, COVID19RF-003, COVID19RF-008, MOH-OFYIRGnov19-0005, and RIE2020 CCGSFPOR20002) (L-FW). Other individual grant supports: ACP Paeds Fund KRDUK18P0200 (KY), NMRC/TA/0059/2017 (JY), NMRC/OFLCG/002/2018 (SA), NMRC/TCR/0015-NCC/2016 (SA), CIRG19may0052 (SA), and MOH-STaR19nov-0002 (SA) are gratefully acknowledged.

## Conflict of Interest

LW, WC, and CT are co-inventors on a patent for the surrogate virus neutralization assay commercialized by GenScript Biotechnology under the trade name cPass.

The remaining authors declare that the research was conducted in the absence of any commercial or financial relationships that could be construed as a potential conflict of interest.

## Publisher’s Note

All claims expressed in this article are solely those of the authors and do not necessarily represent those of their affiliated organizations, or those of the publisher, the editors and the reviewers. Any product that may be evaluated in this article, or claim that may be made by its manufacturer, is not guaranteed or endorsed by the publisher.
