# Supplementary material for: Neutralizing Activity and SARS-CoV-2 Vaccine mRNA Persistence in Serum and Breastmilk After BNT162b2 Vaccination in Lactating Women
Source: Front Immunol. 2022 Jan 11;12:783975. doi: 10.3389/fimmu.2021.783975 (PMC8787073; doi:10.3389/fimmu.2021.783975)
Supplement: Supplementary file 1 [file DataSheet_1.doc]

**List of online-only documents**

1. Supplemental Figure 1. Standard curves generated through the three separate runs on different days using spiked mRNA into non-vaccinated samples

2. Supplemental Table 1. Reverse transcription PCR and quantitative PCR results from maternal serum samples with detectable BNT162b2 mRNA

3. Supplemental Table 2. Reverse transcription PCR and quantitative PCR results from breastmilk samples with detectable BNT162b2 mRNA

**Supplemental Figure 1. Standard curves generated through the three separate runs on different days using spiked mRNA into non-vaccinated samples**

Data points represented as mean ± SEM. Comparisons of the slopes of the curves generated through the three runs indicate that the overall slopes were identical (p=0.9)

**Supplemental Table 1. Reverse transcription PCR and quantitative PCR results from maternal serum samples with detectable BNT162b2 mRNA**

| **Sample ID** | **Dose #** | **Day post vaccination** | **First Run** | | | **Repeat Run** | | | **Ct mean** | **Amount cDNA (fg)** | **Amount in 1ml breastmilk (pg)** | **Amount in 100ml breastmilk (ng)** |
| --- | --- | --- | --- | --- | --- | --- | --- | --- | --- | --- | --- | --- |
|  |  |  | **Ct1** | **Ct2** | **Ct3** | **Ct1**  **repeat** | **Ct2**  **repeat** | **Ct3**  **repeat** |  |  |  |  |
| M003 | 2 | 7 | 37.0 | N | N | 35.8 | 36.5 | 35.5 | 36.2 | 199.5 | 166.2 | 16.6 |
| M004 | 2 | 7 | 35.5 | 35.0 | 35.4 | 37.2 | 35.0 | 35.5 | 35.6 | 60.2 | 50.2 | 5.0 |
| M005 | 1 | 3 | 37.2 | 34.1 | 34.1 | 34.1 | 35.6 | 33.6 | 34.8 | 99.1 | 82.6 | 8.3 |
| M008 | 2 | 6 | 35.7 | 37.5 | N | 37.1 | 37.1 | 37.0 | 36.9 | 139.9 | 116.6 | 11.7 |
| M009 | 2 | 7 | N | 36.0 | 37.3 | 36.1 | N | 34.7 | 36.0 | 219.6 | 183.0 | 18.3 |
| M009 | 1 | 4 | 33.7 | 34.1 | 33.4 | 33.6 | 33.6 | 34.1 | 33.7 | 189.9 | 158.3 | 15.8 |
| M013 | 1 | 3 | 35.7 | 36.1 | 36.9 | 35.0 | 35.2 | 35.3 | 35.7 | 56.4 | 47.0 | 4.7 |
| M017 | 1 | 2 | 36.1 | 36.0 | 35.6 | 34.4 | 35.7 | 34.9 | 35.5 | 66.2 | 55.1 | 5.5 |
| M019 | 1 | 3 | 34.9 | 36.0 | 36.1 | 34.6 | 37.0 | 34.2 | 35.5 | 288.9 | 240.7 | 24.1 |
| M019 | 2 | 5 | 34.4 | 32.8 | 33.5 | 32.9 | 33.9 | 33.5 | 33.5 | 815.1 | 679.3 | 67.9 |
| M020 | 2 | 7 | N | 37.1 | 36.8 | 37.0 | 37.0 | N | 37.0 | 132.9 | 110.7 | 11.1 |
| M022 | 1 | 4 | 33.5 | 33.9 | 34.0 | 34.6 | 35.0 | 34.1 | 34.2 | 570.7 | 475.6 | 47.6 |
| M022 | 2 | 6 | 35.3 | 35.7 | 36.8 | N | 35.2 | 36.0 | 35.8 | 53.6 | 44.6 | 4.5 |
| M024 | 1 | 3 | 35.3 | 33.7 | 33.5 | 34.2 | 34.5 | 35.2 | 34.4 | 126.3 | 105.2 | 10.5 |
| M024 | 2 | 6 | 36.0 | 36.7 | 36.9 | 37.6 | N | N | 36.8 | 29.2 | 24.3 | 2.4 |
| M028 | 1 | 3 | 34.0 | 34.4 | 33.2 | 33.3 | 33.5 | 33.2 | 33.6 | 764.4 | 637.0 | 63.7 |
| M028 | 2 | 7 | 34.5 | 35.0 | 35.8 | 34.4 | 33.8 | 34.0 | 34.6 | 467.4 | 389.5 | 39.0 |
| M029 | 1 | 3 | N | 35.9 | 35.2 | 37.0 | 37.1 | N | 36.3 | 187.9 | 156.5 | 15.7 |
| M031 | 2 | 7 | 37.3 | 36.9 | N | N | 37.2 | 35.5 | 36.7 | 152.2 | 126.8 | 12.7 |
| M033 | 1 | 2 | 35.1 | 36.9 | 35.2 | 35.1 | 34.9 | 36.3 | 35.6 | 275.7 | 229.7 | 23.0 |

N = not detected or melt curve is not the same as the standard curve; Ct=cycle threshold

Only those participants with a minimum of 4 positive out of 6 technical replicates are included

**Supplemental Table 2. Reverse transcription PCR and quantitative PCR results from breastmilk samples with detectable BNT162b2 mRNA**

| **Sample ID** | **Dose #** | **Day post vaccination** | **First Run** | | | **Repeat Run** | | | **Ct mean** | **Amount cDNA (fg)** | **Amount in 1ml breastmilk (pg)** | **Amount in 100ml breastmilk (ng)** |
| --- | --- | --- | --- | --- | --- | --- | --- | --- | --- | --- | --- | --- |
|  |  |  | **Ct1** | **Ct2** | **Ct3** | **Ct1**  **repeat** | **Ct2**  **repeat** | **Ct3**  **repeat** |  |  |  |  |
| M003 | 1 | 1 | 34.5 | 34.3 | 34.8 | 34.5 | 35.0 | 37.2 | 35.0 | 85.5 | 71.2 | 7.1 |
| M014 | 1 | 3 | 37.1 | 37.1 | 36.0 | 35.5 | 38.0 | 36.8 | 36.8 | 51.2 | 42.7 | 4.3 |
| M027 | 2 | 1 | 34.3 | 34.4 | 36.0 | 34.0 | 34.4 | 34.3 | 34.6 | 201.8 | 168.2 | 16.8 |
| M027 | 2 | 3 | 35.4 | 35.3 | 36.3 | 36.4 | 36.0 | N | 35.9 | 89.7 | 74.8 | 7.5 |
| M034 | 2 | 1 | N | N | 36.5 | 35.8 | 36.1 | 36.0 | 36.1 | 77.3 | 64.4 | 6.4 |

N = not detected or melt curve is not the same as the standard curve; Ct=cycle threshold

Only those participants with a minimum of 4 positive out of 6 technical replicates are included
